# Hospital experiences and medical traumatic stress in adults with spina bifida

**DOI:** 10.1177/18758894251333917

**Published:** 2025-04-21

**Authors:** Ellen Fremion, Nora Deibler, Juliana Abel, Monique Ridosh

**Affiliations:** 1 Department of Internal Medicine, Baylor College of Medicine, Houston, TX, USA; 2 Department of Pediatrics, Baylor College of Medicine, Houston, TX, USA; 3 Physician Assistant Program, Baylor College of Medicine, Houston, TX, USA; 4 Family and Community Health Nursing Department, Marcella Niehoff School of Nursing, Loyola University, Maywood, IL, USA

**Keywords:** spina bifida, medical traumatic stress, care experience

## Abstract

**Purpose:**

This study examined hospital and emergency department (ED) experiences of adults with spina bifida (SB), medical traumatic stress (MTS) and participant characteristics including anxiety, depression, post-traumatic stress disorder (PTSD) symptoms, and resiliency scores.

**Methods:**

Adults with SB who had a hospital or ED encounter within the last five years were recruited from a medical home clinic and completed a structured interview and validated questionnaires. Narrative inquiry was used and descriptive analyses were conducted. MTS scores were reported per participant characteristics and emotional health questionnaire score counts and percentages.

**Results:**

Twenty-five adults with SB representing 37% of eligible patients were recruited. Most participants were positive for at least one MTS symptom cluster. There was an increased number of MTS symptom clusters for participants with higher depression, anxiety, and PTSD scores, and decreased MTS symptom clusters for those with increased resilience. The narrative analysis yielded three distinct themes: a negative hospital environment, SB-related condition concerns and complications, and positive support when in the hospital.

**Conclusion:**

Further research with a larger study population is necessary to examine the extent to which MTS is present in individuals with SB of all ages, to understand how MTS symptoms may change with age and experience, and to identify risk and protective factors for emotional health in the presence of MTS. However, this study identified opportunities for improving healthcare experiences for this patient population, including facilitating communication, promoting self-advocacy, self-efficacy, resilience, and familial support which can be implemented for all age groups.

## Introduction

While post-traumatic stress disorder (PTSD) is often associated with events such as combat, assault, and natural or human-made disasters, individuals who have experienced traumatic medical events can also have symptoms of PTSD.^1^ Medical trauma is the experience of traumatic stress resulting from negative interactions with the medical system. These experiences can include invasive procedures (e.g., surgeries), new diagnoses (e.g., cancer), and conflict with healthcare professionals.^2^ As in PTSD, the symptoms of medical traumatic stress (MTS) include psychological symptoms such as intrusive thoughts, re-experiencing the event, avoidance behavior, irritability, anxiety, and depression, as well as somatic symptoms such as insomnia, headaches, abdominal pain, and fatigue.^2^ In children with chronic conditions, those identified as having MTS were more likely to have missed school days and work days for their parents, lower quality of life scores, home opioid use, and negative psychological and physical health outcomes.^3,4^

People with spina bifida (SB) have lifelong, significant, and repeated interactions with the healthcare system that may pose an increased risk for MTS. Most individuals with SB live with neurologic, urologic, musculoskeletal, and gastrointestinal comorbidities that require multiple, and sometimes emergent, medical interventions that can be emotionally and financially stressful for them and their families.^5^ There is one published study evaluating MTS in the parents of children with SB, but MTS has yet to be directly evaluated in children or in adults with SB and thus the prevalence of MTS in SB as well as evidence-based interventions to prevent or remedy MTS throughout the lifespan are lacking.^6^ Additionally, resilience, “the active and dynamic process through which a person adaptively overcomes a stressful or difficult situation or recovers swiftly from a period of ill-health,” has been shown to mitigate the longitudinal effects of traumatic or stressful events.^7^ While resilience has been studied in adults with SB in comparison to the general population and to understand individual characteristics associated with higher or lower resilience, it has not been studied in the context of MTS in SB.^8–10^

This study examined hospital and emergency department (ED) experiences of adults with SB and the co-occurrence of MTS symptoms with PTSD symptoms, anxiety, depression, and resilience. Based on previous studies, demographic factors (race/ethnicity, insurance), increased condition severity (higher level of SB lesion), and increased emotional health morbidity (higher anxiety or depression questionnaire scores, and/or lower resiliency scores) were hypothesized to co-exist with MTS symptoms.^11,12–16^

## Methods

### Study design

The Consolidated Criteria for Reporting Qualitative Research (COREQ) guidelines were used for the multi-method study design and reporting.^17^ Data were recorded using Research Electronic Data Capture (REDCap), a secure, web-based database hosted by academic institutions.^11,18^ Interviews were conducted and recorded using the institution's HIPPA-compliant Zoom video conferencing platform (Version: 5.16.10), and participant responses to the interview and survey questions were recorded and transcribed into REDCap. Finally, demographic and SB condition data were collected from the electronic health record.

#### Interview questions

Participants were asked to recall and describe a time when they were in the ED or admitted to the hospital. Then, they were asked whether the experience was stressful or frightening, and if yes, how so. If not, they were asked to describe how they felt. Next, they were asked what changes could be made to the healthcare environment, and how their healthcare provider, family or friends could have helped, to make the experience better. Finally, the participant was asked what this experience taught them about themselves, or if the experience had changed their perspective on their health.

#### MTS symptom measurement

As there are no validated MTS scales for adults with SB, MTS was assessed using the Pediatric Medical Traumatic Stress measure developed to assess MTS in parents of children diagnosed with SB.^6^ This assessment was selected as questions specifically addressed SB and there have been no MTS assessments developed for or used in adults with SB. Of note, no survey wording needed to be modified to be applicable to adults. The 17-question survey assesses for the MTS cluster symptoms of intrusion, avoidance, and increased arousal using a 4-point Likert scale to determine how frequently the symptom is experienced; a score of one represents seldom or never, and a score of four represents nearly always. Scores of 1–2 indicate that the symptom is not present, while scores of 3–4 indicate the symptom is present. Regarding the symptom clusters, the participant was considered positive for intrusion if they had one or more symptoms, avoidance if they had three or more symptoms, and increased arousal if they had two or more symptoms.

#### PTSD, depression, anxiety, and resilience measurements

PTSD was assessed using the Posttraumatic Stress Disorder Checklist for DSM-5 (PCL-5), a 20-question survey inquiring about the degree of bother of PTSD symptoms over the last month using a 5-point Likert scale; a score of zero indicates not at all, and a score of four indicates extremely.^12^ An overall score of 32 out of 80 indicates probable PTSD.

Depression was assessed using the Patient Health Questionnaire-9 (PHQ-9), a 9-question survey asking the frequency of depression symptoms over the last two weeks using a 4-point Likert scale from (0) “not at all” to (5) “nearly every day”.^13^ Higher overall scores indicate more severe depression (i.e., <5 no depression, and 20–27 severe depression). If the participant indicated on the PHQ-9, or at any point during the interview, that they were experiencing suicidal ideation, the Columbia-Suicide Severity Rating Scale was administered, and the clinic physician and social worker were informed to follow up immediately.^14^

Anxiety was assessed using the General Anxiety Disorder-7 scale (GAD-7), a 7-question survey about the frequency of anxiety symptoms over the last two weeks using a 4-point Likert scale from (0) “not at all” to (3) “nearly every day”.^15^ Higher overall scores indicate more anxiety (i.e., < 4 minimal anxiety and 15 -21 severe anxiety).

Resilience was assessed using the Connor-Davidson Resilience Scale (CD-RISC-10), a 10-question survey asking respondents to rate how well they were equipped to bounce back after stressful events over the last month using a 5-point Likert scale from (0) “not true at all” to (4) “always true”.^16^ A higher score on this survey indicates increased resilience (total scores ranged from 0–40).

#### Demographic and clinical variables

The demographic data collected included age, sex, race, ethnicity, and insurance. The condition data were based on variables collected for the National Spina Bifida Patient Registry including type of SB (myelomeningocele or lipomyelomeningocele), functional level of lesion based on the Hoffer scale, and ambulatory status.^19,20^

### Study population

After receiving Institutional Review Board approval, participants were identified using the clinical record then recruited and consented. Adults with SB (aged 19 and above) were eligible to participate if they were established patients with an academic medical home for adults with intellectual and developmental disabilities, were seen between 2022 and July 30, 2024, and had an ED or hospital encounter in the last five years. Patients were excluded if they did not have myelomeningocele or lipomyelomeningocele, were unable to consent due to intellectual disability, did not have an ED or hospital encounter within the last five years, or were hospitalized for obstetrics delivery only. Patients were asked to participate during clinic visits or via patient portal messaging. Those patients who agreed to participate were contacted by a trained interviewer to give verbal consent for the study and to complete an interview via video conferencing. The interview was recorded, then transcribed into REDCap. The participants’ demographic and clinical data were also recorded in REDCap.

### Data analyses

#### Qualitative analysis

Interview responses were de-identified and reviewed by two authors to identify themes and develop a coding scheme using narrative inquiry. Narrative inquiry introduces “retrospective meaning making” using past experiences. The interpretive process included identifying emotions, thoughts, and interpretations as expressed by the participant's responses. This process guided the synthesis of data to represent the participants stories. Each response was independently coded, and discrepancies were resolved through consensus. Confirmability and dependability were addressed by review of the results with two co-authors not involved in conducting interviews. Saturation was determined as the point when additional interviews did not lead to new themes but provided more exemplars and therefore confirmed saturation. Exemplar quotes were selected to give voice to the participant's experiences.

### Quantitative analysis

As this study was exploratory, descriptive analysis was used. Participant demographics and clinical factors were described using percentages and means with standard deviations. Questionnaire scores were calculated and reported as means with standard deviations and/or counts and percentages per their instructional guidelines. Finally, MTS symptom cluster frequencies were categorized by questionnaire score range counts and percentages. Descriptive statistics were used to summarize findings.

## Results

Twenty-five adults with SB agreed to participate from July 2023- July 2024, representing 37% of the eligible clinic patients. Table 1 shows the distribution of demographic data and SB clinical characteristics. Hospital-related experiences were reported as stressful and/or frightening by most participants (23 out of 25). Their experiences are synthesized below followed by a descriptive analysis of anxiety, depression, post-traumatic stress symptoms, and resiliency.

**Table 1.** Descriptive characteristics of study participants.

Characteristics	Total N = 25 n (%)
Median Age (with range)	29 (19-44)
Sex	
Female	21 (84%)
Male	4 (16%)
Race/Ethnicity	
African American/Black	7 (28%)
Asian/Pacific Islander	1 (4%)
White, Hispanic/Latino	12 (48%)
White, Non-Hispanic/Latino	5 (20%)
Insurance	
Private	4 (16%)
Medicaid	14 (56%)
Private + Medicaid	3 (12%)
Medicare + Medicaid	4 (16%)
SB Type	
Myelomeningocele	22 (88%)
Non-myelomeningocele	3 (12%)
Level of Lesion	
Thoracic	7 (28%)
Lumbar	16 (64%)
Sacral	2 (8%)
Ambulatory Status	
Community ambulator	7 (28%)
Home only ambulator	4 (17%)
Non-ambulator	14 (56%)

**Table 2.** Hospital and emergency department experience themes and response examples.

Themes	Tabulations	Example Responses
**Negative hospital environment**		
**Feeling unheard**	5	“I felt that even when I was an old enough kid to know what I was talking about, they would still listen to my parents over me. They would only listen to my dad and not me even when I was saying something was wrong.”
**Insufficient and inconsiderate communication from the healthcare team**	6	“I feel like that my care team wasn't the most forthcoming… I felt like my questions were a burden, and if I needed answers on anything, it was, “why are you being problematic?” “One of the nurses there kind of frightened me because they were saying because of how frequent urinary tract infections I get, one day my body might become resistant to the antibiotics, so that was frightening to me.”
**Unwelcoming adult hospital environment**	5	“When they sent me to the hospital it was very lonely and dark… In the hospital everything was very sad. It was a very dark experience. It was the first time I was in a hospital for adults, so it was very emotional to be there on my own. I was there for two days.”
**Frustration with care delays**	7	“I was told by a surgeon that day that I had to go to the hospital…I went that night, I did everything I was supposed to do and called him. However, I and the ER staff could not get a hold of him. I ended up spending almost all day in the ER. When I finally got released, it was because the surgical site was not “in their mind” medically necessitating an admission.”
**Total**	**23**	
**SB-related condition concerns and complications**		
**Adult providers lacking knowledge about SB care**	9	“If I have a doctor who thinks they know better because they went to medical school, I get upset. Because I know my body more than you know your note, so don't try to throw ‘I went to medical school’ in my face.” “They [healthcare providers] just really didn't have a lot of experience working with people with Spina Bifida and you know, [those with] chronic childhood illness that are now adults. [They] just didn't have the knowledge and supports in place to help me.”
**Multiple lifetime hospitalizations and SB health complications**	25	“I have stress, because you know, it's almost like, Okay, well, is something going to come back? You know? And then of course I always wonder to myself, what else with me having spina bifida, what else has been affected, you know, other than just my bladder and my bowels?”
**Pain**	10	“The more pain I was caused because of the issues the more I felt like they left PTSD.”
**Urologic Concerns**	11	“The most stressful [ER experience I’ve had], my urethra had swollen and the catheter wouldn't go in. I started trying to self-cath at 10 PM, and by 12 AM, I still wasn't able to.” “I had surgery for cystoplasty/augmentation. They had to open me up. That was kind of scary because they left a really big scar that I can see.”
**Skin/bone infections**	3	“Getting my toe amputated off was a big change for me because, even though I am disabled and, in a wheelchair, it is my big toe.”
**Shunt/neurosurgery**	5	“The last time, my back was hurting in my head. It ended up being my shunt. I don’t know what was wrong but my shunt had broken. I knew right then and there that I was going into surgery. I was like “Oh Lord” let me get prepared, because I know what is going to happen. I am used to going in and going into surgery. I am used to that, but I am tired of it. It is just a lot. I get frustrated, it is just too much. They have to shave my head to get to the part. I really hate that part.”
**Anxiety about procedures and illness severity**	16	“Getting constantly pricked when they can't find my veins was very stressful for me.” “[I was] in ICU for a week, having those issues going on, my anxiety level was like through the roof.”
**MTS symptom clusters**	11	“I don't like to look at myself in the mirror that much anymore. I avoid looking at the scar.” “So number one I don't like needles, because as a kid I was stuck a LOT of times in one arm with a needle, and so it's very traumatic for me, even like today, right now, … going to the hospital and having to do blood, knowing that I have to do blood, I get major anxiety, and I try not to think about it.” “I haven’t talked about [that hospital stay] since then. Sometimes playing it back makes me a little anxious and a little emotional.”
**Total**	90	
**Positive supports when in the hospital**		
**Self-Advocacy/Self-Efficacy**	5	“As a peer mentor, I use my experiences to help my mentees so they don't have to go through the same thing and can learn from my experiences.” “It made me really proud of myself to know that I am well equipped to say the things I did and know what I know.”
**Resilience**	10	“I knew that I was going to be good even though I was scared just a little bit.” “I had many falls in my childhood, and I have gotten myself back up, physically and metaphorically.” “It makes me stronger to deal with this - getting in and out of my chair, doing things on my own, I am used to it.”
**Family Support**	13	“My mom is always there to help; she has been everything from day one.” “Honestly, I have to have somebody with me. If I go to the ER or something so like. For example, my mom or somebody will have to go with me so that way I can keep calm and make sure that I'm good, you know.”
**Positive Patient-Provider Communication**	9	“Every doctor on your team including your surgeon should come and see you at least every other day. The staff and surgeon should communicate with each other every day. “ “Since they told me what was wrong and what was going on, it made me feel better.” “The most recent time I went to the ER for my urinary tract infection. The staff there were very knowledgeable on the process of my UTIs that I frequently get, and they were also very friendly and patient with me.”
**Positive care outcome**	10	“The teams I have had thus far in my life have really helped.” “I had a good experience because I had good doctors and nurses taking care of me.”
**Total**	47	

SB: Spina bifida; MTS: medical traumatic stress.

### ED and hospital experience themes

Narrative analysis of the interview responses yielded three main themes: *negative hospital environment, SB-related condition concerns and complications,* and *positive supports when in the hospital.* Exemplar quotes were listed in Table 2 including themes and subthemes. The first theme of a negative hospital environment reflected aspects of communication, the hospital system care coordination, and the physical environment of the hospital. As adults, participants felt unheard when communicating something was wrong to health professionals (e.g., “they would still listen to my parents over me”). Participants reported feeling their questions were a “burden” and “problematic.” One participant recalled a frightening communication about how their frequent UTIs might lead to their body becoming resistant to antibiotics in the future. Several participants reported feeling unwelcome in the hospital environment, one referring to it as a “lonely and dark” place. Further many experienced frustrations with care delays such as when doing “everything I was supposed to do” when reporting complications after surgery that led to “spending almost all day in an emergency department.” SB-related condition concerns and complications were prevalent and included both physical and emotional aspects of the SB care experience. Participants felt adult providers lacked knowledge about SB care and support they needed stating “I know my body more” and that providers “didn't have a lot of experience working with people with spina bifida” who were now adults. All participants had experienced multiple hospitalizations beyond the period necessary for inclusion in this study (previous 5 years) and health complications related to their spina bifida. The chronic nature of the condition over time was stressful as the following participant shared: “I have stress, because you know, it's almost like, okay, well, is something going to come back? You know? And then of course I always wonder to myself, what else with me having spina bifida, what else has been affected, you know, other than just my bladder and my bowels?” Participants reported experiencing pain, and one participant linked pain from issues over time with PTSD. Two accounts describe urological concerns as “most stressful” and “scary”: “The most stressful [ER experience I've had], my urethra had swollen, and the catheter wouldn't go in. I started trying to self-cath at 10 PM, and by 12 AM, I still wasn't able to.” “I had surgery for cystoplasty/augmentation. They had to open me up. That was kind of scary because they left a really big scar that I can see.” A few participants shared their experience with skin/bone infections. One participant shared being changed by an amputation “even though I am disabled and in a wheelchair, it is my big toe.” The anticipation of requiring shunt/neurosurgery care was concerning as in the case of shunt revision one participant exclaimed… “Oh Lord let me get prepared, because I know what is going to happen. I am used to going in and going into surgery. I am used to that, but I am tired of it. It is just a lot. I get frustrated, it is just too much. They have to shave my head to get to the part. I really hate that part.” More than half of participants shared their anxiety about needed procedures and illness severity. Specifically, saying “getting constantly pricked when they can't find my veins was very stressful” and for one in the ICU for a week… “having those issues going on, my anxiety level was like through the roof.” Accounts of MTS symptom clusters (n = 11) detailed instances of intrusion, avoidance, and increased arousal such as the following: “I don't like to look at myself in the mirror that much anymore. I avoid looking at the scar.” “So number one I don't like needles, because as a kid I was stuck a LOT of times in one arm with a needle, and so it's very traumatic for me, even like today, right now, … going to the hospital and having to do blood, knowing that I have to do blood, I get major anxiety, and I try not to think about it.” “I haven't talked about [that hospital stay] since then. Sometimes playing it back makes me a little anxious and a little emotional.” Contrasting these experiences, the third theme focused on evidence of positive supports participants acknowledged or recommended to improve the hospital experience. One participant said they used their experiences to help others (i.e., peer mentoring) and another felt proud they were “well equipped to say the things I did and know what I know.” They shared about their ability to speak up for ones needs (i.e., self-advocacy) and their belief in abilities to achieve goals in caring for themselves (i.e., self-efficacy). Almost half (n = 10) spoke of developing resilience such as knowing they were “going to be good even though I was scared just a little bit.” Evidence of the “bouncing back” characteristic of resilience is noted in this account: “I had many falls in my childhood, and I have gotten myself back up, physically and metaphorically.” Another said it made them “stronger to deal with this-getting in and out of my chair, doing things on my own, I am used to it”. Many of the participants (n = 13) shared the value and presence of family support such as being accompanied by a parent in the hospital setting. One participant shared being accompanied (by parent or other) as necessary to “keep calm and make sure that I'm good, you know.” In contrast to poor communication previously described several experiences included positive patient/provider communication and highlighted the importance of regular communication and how they felt better when they were told “what was wrong and what was going on.” One acknowledged very knowledgeable staff who were “friendly and patient.” Positive care outcomes were attributed to health care teams that have helped and the “good doctors and nurses taking care of me.”

### MTS scores

Table 3 shows MTS scores grouped by symptom clusters and the distribution of positive symptom clusters. The mean score for intrusion was 9.2 out of a possible 20, the mean score for avoidance was 12.2 out of a possible 28, and the mean score for arousal was 10.8 out of a possible 20. Seven participants (28%) were negative for all three clusters, eight (32%) were positive for one cluster, seven (28%) had intrusion and arousal, one (4%) had intrusion and avoidance, and three (12%) had all three symptom clusters.

**Table 3.** Mean PMTS scores per symptom clusters and positive symptom cluster frequency.

Question	Mean score	Standard deviation
**Intrusion (Total Mean Score: 9.2)**		
• Do you have recurrent, intrusive recollections of events in your thoughts?	2.0	0.95
• Do you have recurrent, intrusive recollections of events in nightmares?	1.4	0.71
• Do you have recurrent, intrusive recollections of events in flashbacks?	1.8	0.65
• Do you have intense feelings of distress when confronted with anything that has/had to do with the SB situation?	2.0	0.95
• Do you have physical reactions (e.g., respiration, aching muscles, stomach, and/or head) when confronted with anything that has/had to do with the SB situation?	2.0	0.95
**Avoidance (Total Mean: 12.2)**		
• Do you try to avoid anything to do with SB?	1.4	0.83
• Do you seek distraction to avoid thinking about the SB situation?	1.9	0.88
• Do you have trouble recalling important aspects of the SB situation?	1.8	1.03
• Do you have markedly diminished interest in significant social activities?	2.0	0.75
• Do you feel detached or estranged from the world around you?	2.0	0.88
• Do you feel unable to feel affection or love for others?	1.4	0.58
• Do you have a sense that your future had been foreshortened?	1.6	0.71
**Arousal (Total Mean: 10.8)**		
• Do you have difficulty falling or staying asleep?	2.4	0.87
• Do you feel more irritated than usual/have outbursts of anger?	2.0	1.02
• Do you have difficulty concentrating?	2.2	1.02
• Are you in a state of constant alertness?	2.2	1.18
• Are you more nervous than usual?	2.0	0.80

Clusters	N = 25
None	7 (28%)
Intrusion alone	6 (24%)
Arousal alone	1 (4%)
Intrusion + Avoidance	1 (4%)
Intrusion + Arousal	7 (28%)
Intrusion + Avoidance + Arousal	3 (12%)

PMTS: Pediatric Medical Traumatic Stress measure; SB: spina bifida.

### Emotional health questionnaire scores

Table 4 shows the emotional health questionnaire scores. Based on PCL-5 scores, seven participants (28%) had PTSD symptoms. Based on GAD-7 scores, seven participants (28%) had mild anxiety, five (20%) had moderate anxiety, and four (16%) had severe anxiety. Based on PHQ-9 scores, 14 participants (56%) had mild-moderate depression, and one (4%) had moderate-severe depression. Based on CD-RISC-10 scores, one participant (4%) scored between 0–9 (indicating the lowest resilience), 15 (60%) were in the middle ranges, and eight (32%) scored 30–40 (indicating the highest resilience).

**Table 4.** Emotional health scores for study participants by data collection instrument.

Scale	Mean score (standard deviation)	Score categories	Total N = 25 n (%)
PCL-5	21.9 (15.8)	<32: no PTSD	18 (72%)
		32+: PTSD	7 (28%)
GAD-7	7.0 (5.6)	<5: none/minimal anxiety	9 (36%)
		5-9: mild anxiety	7 (28%)
		10–14: moderate anxiety	5 (20%)
		15+: severe anxiety	4 (16%)
PHQ-9	7.1 (4.7)	<5: none/minimal depression	10 (40%)
		5–14: mild-moderate depression	14 (56%)
		15+: moderately severe-severe depression	1 (4%)
CD-RISC-10	24.4 (8.5	0–9	1 (4%)
		10–19	5 (20%)
		20–29	11 (44%)
		30–39	8 (32%)

PCL-5: Post-Traumatic Stress Disorder Checklist for DSM-5; GAD-7: General Anxiety Disorder-7; PHQ-9: Patient Health Questionnaire-9; CD-RSIC-10: Connor-Davidson Resilience Scale 10-Item; PTSD: post-traumatic stress disorder.

### MTS scores by participant characteristics and emotional health questionnaire scores

#### MTS scores by participant characteristics

Table 5 shows the participant characteristics for MTS score clusters. Seven (33%) females, but no males, were negative for MTS symptom clusters. More than half (58%, n = 4) of Black participants and Asian/Pacific Islander participants, 66% (n = 8) of Hispanic/Latino participants, and 100% (n = 5) of White, non-Hispanic/Latino participants scored positive for at least one symptom cluster. In relation to insurance categories, most had one to two positive symptom clusters. Those with Medicaid only had the highest percentage of negative scores (46%, n = 6), and the Medicare + Medicaid group had the highest percentage of three positive symptoms (20%, n = 2). Table 6 shows the SB condition characteristics compared to PMTS scores. Those with myelomeningocele ranged from 0–3 symptom clusters, and non-myelomeningocele 1–2 symptom clusters. All three participants with three symptom clusters had thoracic level SB and were non-ambulatory. MTS symptom clusters varied according to clinical characteristic categories.

**Table 5.** Distribution of positive PMTS symptom clusters by participant demographic characteristics.

Demographic Characteristics		Number of positive PMTS cluster categories			
		No symptom clusters	1 symptom cluster	2 symptom clusters	3 symptom clusters
Age	≤ 29 years old (n = 14)	4 (29%)	4 (29%)	5 (36%)	1 (7%)
	≥ 30 years old (n = 11)	3 (27%)	3 (27%)	3 (27%)	2 (18%)
Sex	Male (n = 4)	0 (0%)	1 (25%)	2 (50%)	1 (25%)
	Female (n = 21)	7 (33%)	6 (29%)	6 (29%)	2 (9%)
Race/ethnicity	African American/Black (n = 7)	3 (43%)	2 (29%)	2 (29%)	0 (0%)
	Asian/Pacific Islander (n = 1)	0 (0%)	1 (100%)	0 (0%)	0 (0%)
	White, Non-Hispanic/Latino (n = 5)	0 (0%)	0 (0%)	3 (60%)	2 (40%)
	White, Hispanic/Latino (n = 12)	4 (33%)	4 (33%)	3 (25%)	1 (8%)
Insurance	Private Only (n = 5)	1 (20%)	1 (20%)	2 (40%)	1 (20%)
	Medicaid Only (n = 13)	6 (46%)	2 (15%)	5 (38%)	0 (0%)
	Private + Medicaid (n = 3)	0 (0%)	2 (66%)	1 (33%)	0 (0%)
	Medicare + Medicaid (n = 4)	0 (0%)	2 (50%)	0 (0%)	2 (50%)

PMTS: Pediatric Medical Traumatic Stress measure.

**Table 6.** Distribution of positive MTS symptom clusters per SB condition category.

SB Characteristic		Number of positive MTS symptom clusters			
		No symptom clusters	1 symptom cluster	2 symptom clusters	3 symptom clusters
Type of SB	Myelomeningocele (n = 22)	7 (32%)	6 (27%)	6 (27%)	3 (14%)
	Non-myelomeningocele (n = 3)	0	1 (33%)	2 (66%)	0
Level of Lesion	Thoracic (n = 7)	2 (29%)	0	2 (29%)	3 (43%)
	Lumbar (n = 16)	5 (31%)	6 (38%)	5 (31%)	0
	Sacral (n = 2)	0	1 (50%)	1 (50%)	0
Ambulatory Status	Community ambulator (n = 7)	0	4 (57%)	3 (43%)	0
	Home-only ambulator (n = 4)	3 (75%)	0	1 (25%)	0
	Non-ambulatory (n = 14)	4 (29%)	3 (21%)	4 (29%)	3 (12%)

MTS: Medical traumatic stress; SB: spina bifida.

#### MTS scores by emotional health scores

Table 7 shows emotional health questionnaire scores in relation to MTS scores. All participants who were negative for PTSD (n = 19) also had no positive MTS symptom clusters, whereas all who were positive for PTSD (n = 6) were positive for two or more MTS symptom clusters. All patients with severe anxiety (n = 4) or moderate anxiety (n = 4) also scored positive for two or more symptom clusters. Nine (90%) of the participants with none/minimal depression scores had 0–1 positive MTS symptom cluster, whereas nine (64%) of the participants with mild-moderate depression scores had 2–3 positive clusters. The participant who had a severe depression score also had three positive MTS symptom clusters. Finally, MTS symptom cluster counts were variable across CD-RISC-10 scores.

**Table 7.** Distribution of PMTS symptom clusters by emotional health data for study participants.

Emotional health questionnaire score		Number of Positive PMTS Symptom Clusters			
		No symptom clusters	1 symptom cluster	2 symptom clusters	3 symptom cluster
PCL 5	<32: no PTSD (n = 19)	7 (37%)	7 (37%)	4 (21%)	1 (5%)
	32+: PTSD (n = 6)	0	0	4 (66%)	2 (33%)
GAD 7	<5: none/minimal anxiety (n = 9)	4 (44%)	4 (44%)	1 (11%)	0
	5-9: mild anxiety (n = 7)	2 (29%)	3 (43%)	2 (29%)	0
	10–14: moderate anxiety (n = 5)	1 (20%)	0	2 (40%)	2 (40%)
	15+: severe anxiety (n = 4)	0	0	3 (75%)	1 (25%)
PHQ 9	<5: none/minimal depression (n = 10)	4 (40%)	5 (50%)	1 (10%)	0
	5–14: mild-moderate depression (n = 14)	3 (21%)	2 (14%)	7 (50%)	2 (14%)
	15+: moderately severe-severe depression (n = 1)	0	0	0	1 (100%)
CD-RISC 10	0–9 (n = 1)	1 (100%)	0	0	0
	10–19 (n = 5)	2 (40%)	0	2 (40%)	1 (20%)
	20–29 (n = 11)	1 (9%)	4 (36%)	5 (45%)	1 (9%)
	30–39 (n = 8)	3 (38%)	3 (38%)	1 (13%)	1 (13%)

PMTS: Pediatric Medical Traumatic Stress measure; PCL-5: Post-Traumatic Stress Disorder Checklist for DSM-5; GAD-7: General Anxiety Disorder-7; PHQ-9: Patient Health Questionnaire-9; CD-RSIC 10: Connor-Davidson Resilience Scale 10-Item; PTSD: post-traumatic stress disorder.

## Discussion

As the life expectancy of individuals with SB increases with improved pediatric medical intervention, advancement in research and evidence-based care is critical for adults with SB.^10,21,22^ Several studies in adults with SB have described increased hospitalization due to SB-related urinary tract infections, pressure injuries with skin/bone infections, device complications and emotional health morbidity compared to the general population, which may pose increased risk for MTS.^23–28^ This study is one of the first to describe ED/hospital experiences and MTS in individuals with SB. Of note, many participants describe multiple stressful ED and hospital experiences across their lifetime making these findings relevant to the care of children and adolescents with SB as well.

### Healthcare experiences and opportunities to mitigate MTS

Narrative analysis of the interview responses identified opportunities to improve the healthcare experience for patients with SB and potentially mitigate MTS. In this study, patient-provider communication contributed to both negative and positive healthcare experiences, a finding not unique to the SB population. As Ha and Longnecker found, health care providers commonly overestimate the effectiveness of their communication with patients, and doctors sometimes discourage patients from asking questions and voicing concerns.^29^ In contrast, patient-centered approaches to communication improve both patient and provider satisfaction, as participants in this study suggested. The impact of positive communication was also noted in a study of patients evaluated for chest pain in the ED, in which provider communication perceived as “good” during the encounter was associated with decreased PTSD symptoms the following week.^30^ Other participants in this study described a negative hospital experience due to the difficulty of transitioning from a pediatric to adult hospital. Similarly, Cox et al. found that patients with SB and their parents felt their pediatric care clinic was superior to their adult care clinic.^26^ In a review, Monaghan et al. highlight the importance of patient-provider communication in establishing trust, promoting positive health outcomes, and improving health care experiences for adolescents and young adults with chronic conditions from childhood.^31^ They additionally offer strategies to improve communication with adolescents and young adults transitioning to adult care.^31^ Further study is needed to evaluate the relationship between MTS in the SB population and patient-provider communication about the hospital experience and transition to adult care.

Participants also voiced the challenges of managing frequent health concerns and unexpected and/or severe exacerbations of their condition. Additionally, they emphasized the stress and anxiety of facing pain, painful procedures, major surgeries, and life-threatening illness. Several described MTS symptoms of avoidance and arousal when recalling these experiences. Similarly, previous research has evaluated the cumulative effect of chronic illness in children and adults with conditions such as cancer, cardiovascular disease, chronic pain, diabetes, arthritis, and cystic fibrosis; the number of stressors (physical, psychological, social) present during a health event, recurrent and unexpected exacerbations, and the presence of parent PTSD symptoms have been associated with increased patient PTSD symptoms.^30,32–34^ Further study is needed to describe the cumulative effect of these experiences when living with spina bifida.

As in other studies, participants in this study described how self-advocacy, self-efficacy, the presence of supportive family, positive communication with the health care team, and resilience helped them face health challenges and improved their care experience.^27,28^ In their family resilience network framework, Rolland and Walsh describe how involving families in care plans, helping families find meaning within the context of disease, and developing a strong collaboration between healthcare team members and families promotes patient and family resilience.^27^ Similarly, Isokääntä et al. provide strategies for increasing resilience and decreasing the risk for MTS in children undergoing surgery and their parents; these include explaining the care plan and why procedures are needed in a way that patients understand, talking with patients about their worries and fears, supporting contact with family and friends, and controlling pain.^28^ Cuneo et al. also provide a comprehensive framework of strategies to prevent, assess for, and intervene in MTS using a trauma-informed approach involving the healthcare team and family.^35^ Such frameworks may be adapted to SB clinical care in inpatient and outpatient settings and used to develop studies to further explore interventions to prevent and reduce MTS in the SB population.

### SB characteristics and emotional health factors

MTS symptoms were present across demographic and SB characteristics, but participants with the highest depression, anxiety, and PTSD score ranges were also positive for two or more MTS symptoms. Previous studies have shown that depression, anxiety, and PTSD are all risk factors for development of further PTSD.^36^ As seen in this study and several other studies, depression, anxiety, and stressful/frightening hospital experiences are common in the SB population, and thus, suggest the potentiality for developing MTS and PTSD.^10,28,37^ However, while previous studies have shown that increased resilience was associated with decreased PTSD, participants in this study with no MTS symptoms ranged across the resilience scores and participants with the highest resilience scores ranged from having no symptoms to 2–3 MTS symptom clusters.^37^ Further study with an expanded sample size is needed to better understand the relationship between resilience and MTS in the SB population.

### Limitations

While sample size was adequate for qualitative analysis, this study was limited by its small sample size of 25 adults with SB from a single clinic for correlational descriptive analyses. Thus, statistical methods to determine associations between variables were not applied, and the study has limited generalizability. While patients were recruited over a year, several declined to participate or did not respond to portal messages. However, this study provides preliminary evidence of the prevalence of MTS and mental health concerns in adults with SB and the protocol can be used for future multicenter studies.

## Conclusions

This study identified multiple opportunities to improve the healthcare experience and potentially mitigate MTS in adults with SB, such as improving patient-provider communication during hospital/ED encounters and transition to adult care, as well as facilitating self-advocacy, self-efficacy, family support, and resilience. While this study found co-occurring MTS and depression, anxiety, and PTSD symptoms, additional studies are needed to fully assess for associations and the temporal relationships between experiences and symptoms. Additional studies should also evaluate how treating depression, anxiety, and PTSD symptoms, and fostering resilience, impact MTS.
