# Corrigendum to “Hospital Experiences and Medical Traumatic Stress in Adults with Spina Bifida”

**DOI:** 10.1177/00952443251357392

**Published:** 2025-08-19

Fremion E, Deibler N, Abel J, Ridosh M. Hospital experiences and medical traumatic stress in adults with spina bifida. *J Pediatr Rehabil Med*. 2025; 18(3). DOI: 10.1177/18758894251333917

The authors contacted the Editor and requested to revise the article as the wrong version had been published.

To correct this error, the article has been revised and republished.

